# A Shared Epithelial Barrier Program Across Mucocutaneous Lichen Planus: A Compartment-Aware Multi-Cohort Transcriptomic Reanalysis

**DOI:** 10.3390/ijms27219534

**Published:** 2026-10-25

**Authors:** Loulwah Alothman, Alaa Alnoor Alameen, Mougina K. Eltahir

**Affiliations:** 1 Department of Oral Medicine and Diagnostic Sciences, College of Dentistry, King Saud University, Riyadh 12371, Saudi Arabia; 2 Department of Pharmacology and Toxicology, College of Pharmacy, King Saud University, Riyadh 11451, Saudi Arabia; aalameen1.c@ksu.edu.sa (A.A.A.);

**Keywords:** lichen planus, oral lichen planus, transcriptome, epithelial barrier, cornified envelope, microarray reanalysis

## Abstract

Oral lichen planus (LP) is widely described as molecularly distinct from cutaneous LP, yet most supporting transcriptomic evidence compares mucosal epithelium with full-thickness skin. We reanalysed four public microarray series covering oral, genital and cutaneous LP (101 arrays) with matched microRNA arrays, using covariate-adjusted models, meta-analysis, cross-platform integration, a within-platform site-by-disease interaction model, pathway and deconvolution scoring and a test of experimentally supported microRNA–target interactions. Eighty-five genes were differentially expressed at both discovery sites, 83 concordantly, dominated by cornified-envelope and keratinisation genes; BPIFC, CDSN and WFDC12 replicated in all four cohorts. A 52-gene barrier score separated lesional from control tissue in the discovery and genital cohorts (AUC 0.98–1.00, partly in-sample), and panels derived at one LP site transferred across study, platform and site (AUC 0.95–0.99), but the score did not reach significance in the only fully independent oral cohort (AUC 0.76, *p* = 0.13). In comparator series, the programme was also induced in psoriasis and partly in cutaneous lupus, but not in atopic dermatitis, eosinophilic oesophagitis or periodontitis, so it is shared rather than LP-specific. Interferon and MYC-target activity and several immune populations were higher in cutaneous LP, but an epithelial:immune compartment proxy showed that cutaneous biopsies were full-thickness whereas mucosal biopsies were epithelium-only, confounding this contrast. Within one platform, 292 genes showed an oral-versus-genital interaction. Neither miR-20a-5p nor miR-34a-5p tracked barrier expression, and across 222,203 supported microRNA–target pair correlations were predominantly positive rather than inverse. Across LP sites, a reproducible epithelial barrier programme is the most consistent shared feature, although it is not specific to LP and its transfer to independent cohorts remains provisional; reported immune divergence cannot be separated from the biopsy compartment.

## 1. Introduction

Lichen planus (LP) is a chronic T-cell-mediated inflammatory disease that affects skin, oral mucosa, genital mucosa, nails and scalp, often in the same patient [1,2]. Oral LP (OLP) is the most persistent phenotype, is frequently refractory to topical therapy, and is classified as an oral potentially malignant disorder, with pooled malignant transformation estimates ranging from 0.44% under strict diagnostic criteria to 2.28% in the highest-quality studies [3,4,5]. Extraoral LP carries a lower but non-zero risk, with vulvar disease accounting for most reported transformations [6]. Whether these clinical differences reflect distinct molecular diseases or a single lichenoid programme expressed in tissues with different barrier architecture, microbial exposure, and hormonal environment remains unresolved [7].

Mechanistic work over the last decade has converged on a cytotoxic interferon axis. Interferon-γ enhances CD8-mediated keratinocyte killing through JAK2/STAT1 in LP skin [8], type I interferon responses trigger the lichenoid cytotoxic attack in single-cell profiles of LP [9], and an interferon-γ-driven murine model reproduces mucosal lichenoid injury [10]. Single-cell and spatial studies have added cellular resolution: cDC2A–CXCL13^+^CD8^+^ T-cell–epithelial crosstalk in cutaneous and mucosal LP [11], functional T-cell exhaustion in OLP [12], unconventional T-cell contributions [13], SFRP2^+^ fibroblasts amplifying inflammation [14], and keratin-17-mediated layer-specific responses detected by spatial proteomics [15]. In parallel, keratinocyte-intrinsic barrier failure has emerged as more than a bystander phenomenon: oral keratinocytes are active immune effectors [16], epidermal differentiation complex genes are transcriptionally rewired in inflamed epithelium [17], and LCE and SPRR family members participate directly in inflammatory signalling rather than only in envelope assembly [18,19,20].

Comparative transcriptomic evidence across LP sites, however, remains thin, and the few available comparisons are constrained by the small, singly analysed public series that dominate the field. Reported oral-versus-cutaneous differences are difficult to interpret because the underlying datasets differ simultaneously in platform, laboratory and, critically, in what tissue was placed on the array: mucosal studies commonly profile separated epithelium, whereas dermatological studies profile full-thickness skin containing the entire inflammatory infiltrate and dermal stroma. Cellular composition is now recognised as a dominant driver of bulk transcriptomic contrasts in inflammatory skin disease [21,22], and deconvolution accuracy depends heavily on reference choice and on cell types missing from the reference [23,24,25]. Any cross-site comparison that ignores compartment is therefore at risk of reporting a sampling artefact as biology. A second open question is specificity: cornified-envelope and terminal-differentiation genes are also dysregulated in psoriasis and atopic dermatitis [26,27], and early skin-focused profiling distinguished LP from these dermatoses mainly by its CXCL9-dominated interferon response [28], so a barrier programme shared across LP sites need not be specific to LP.

We therefore reanalysed, in full, all eligible public LP expression series identified by our GEO search, rather than the small subsets used in the original reports, with four strictly bounded aims: (i) to define, with tiered replication evidence, the transcriptomic programme that is shared by oral, genital and cutaneous LP; (ii) to quantify site divergence in a way that separates disease biology from biopsy compartment, using a within-study, single-platform interaction model as the primary divergence analysis; (iii) to test, in matched tissue microRNA data, whether microRNAs previously nominated from network topology alone are actually associated with the shared barrier programme; and (iv) to test, in public series from other chronic inflammatory skin and mucosal diseases, whether that programme is specific to LP. We deliberately do not attempt to explain clinical heterogeneity, treatment response or malignant transformation from expression data, and we report the microRNA analysis as a negative result.

## 2. Results

### 2.1. Cohorts and Quality Control

**Figure 1:**
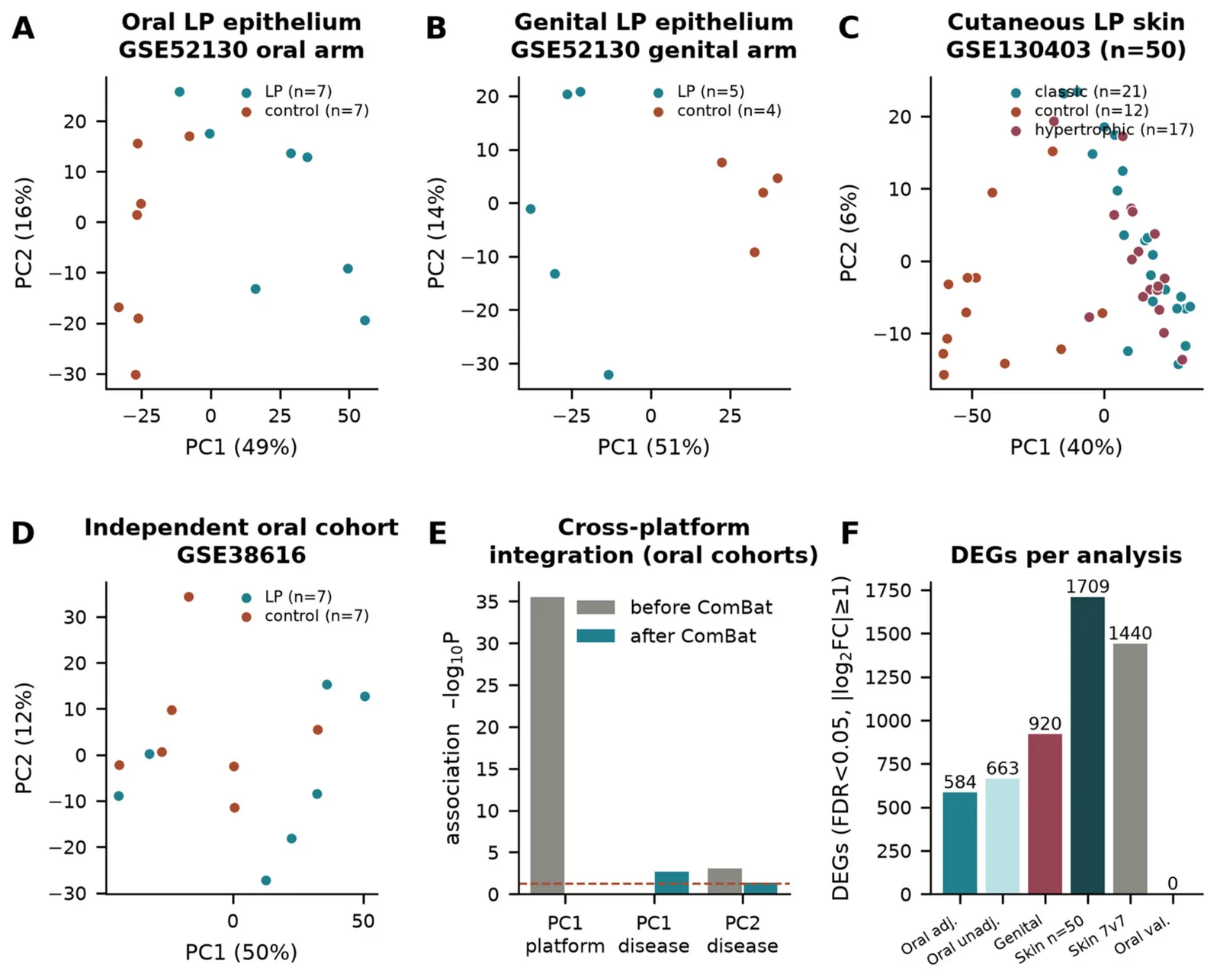
Cohorts, quality control and batch structure. (**A**–**D**) Principal-component analysis of the oral discovery, genital, cutaneous and independent oral cohorts, samples coloured by group. (**E**) Association of PC1 with platform and with disease status in the merged oral matrix before and after ComBat correction (−log_10_P). (**F**) Differentially expressed gene counts by cohort and contrast (FDR < 0.05, |log_2_FC| ≥ 1).

The four analysed series comprise 101 arrays across three anatomical sites (Table 1, Figure 1). Principal-component analysis separated lesional from control samples in the oral discovery cohort (PC1 versus disease, *p* = 0.0001; PC1 versus sex, *p* = 0.13), the genital arm (*p* < 0.001) and the cutaneous cohort (*p* < 0.001), whereas in the independent oral cohort GSE38616 no principal component was associated with disease status (PC1, *p* = 0.24), an early indication of its limited power (Figure 1A–D, Table S2). Two cutaneous arrays were flagged as correlation outliers but were retained, since their removal did not alter the leave-one-out stability of the shared signature. Merging the two oral cohorts without correction produced a matrix whose first component was almost entirely platform-driven (PC1 versus platform, *p* = 3.1 × 10^−36^; PC1 versus disease, *p* = 0.90); after ComBat, platform association was abolished, and disease association appeared on PC1 (*p* = 0.99 and *p* = 0.002, respectively; Figure 1E).

### 2.2. Differential Expression by Site, and the Effect of Design Choices

**Figure 2:**
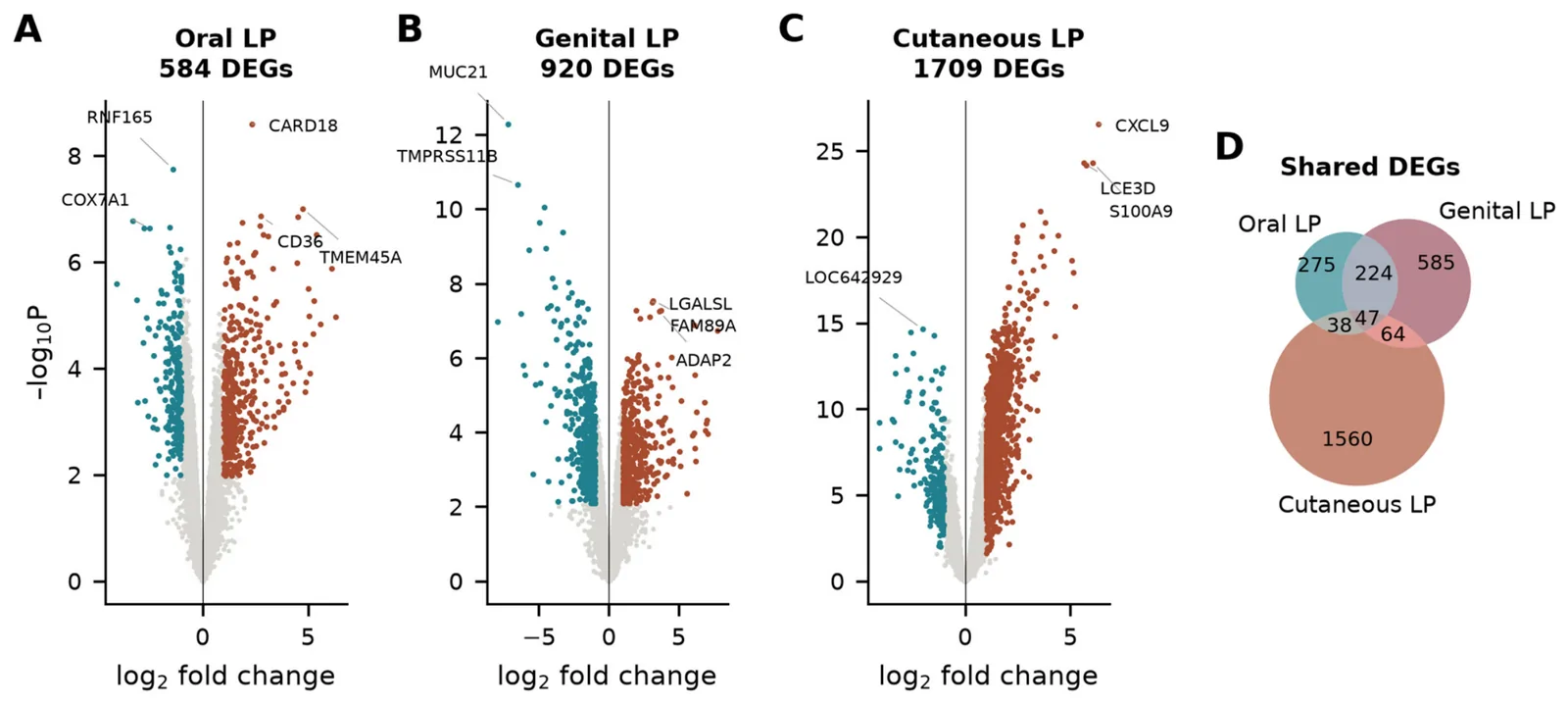
Differential expression landscape. Volcano plots for (**A**) oral LP epithelium (sex-adjusted), (**B**) genital LP epithelium, and (**C**) cutaneous LP skin, with the number of significant genes annotated, and (**D**) a Venn diagram of the three significant gene sets.

In oral LP epithelium, 584 genes were differentially expressed (367 up, 217 down) with sex adjustment, compared with 663 without adjustment, so 12% of the unadjusted signal was attributable to an unmodelled covariate. Genital LP yielded 920 differentially expressed genes and cutaneous LP 1709 (1522 up, 187 down) in the full series; down-sampling the cutaneous cohort to 7 versus 7 still yielded 1440 genes, indicating that the larger cutaneous signal is not simply a sample-size effect. Classic and hypertrophic cutaneous lesions differed from normal skin by 1792 and 1689 genes, respectively, but did not differ from each other at any gene (0 genes at FDR < 0.05), justifying their pooling (Figure 2, Tables S3–S8).

The independent oral cohort GSE38616 yielded no genes at FDR < 0.05 genome-wide (Table S9). This is an important negative control for the design of earlier work: a 7-versus-7 cohort of this kind cannot serve as genome-wide validation, and it is used here only for targeted, pre-specified replication of individual signature genes and of the barrier score. Random-effects meta-analysis of the two oral cohorts gave 198 significant genes with 1877 genes showing significant heterogeneity, and ComBat-based analysis of the merged matrix gave 211 genes; the two approaches overlapped in 151 genes (Jaccard 0.585), so the shared oral signal is reproducible under two structurally different integration strategies (Tables S10 and S11).

### 2.3. A Tiered, Replication-Graded Epithelial Barrier Signature

**Figure 3:**
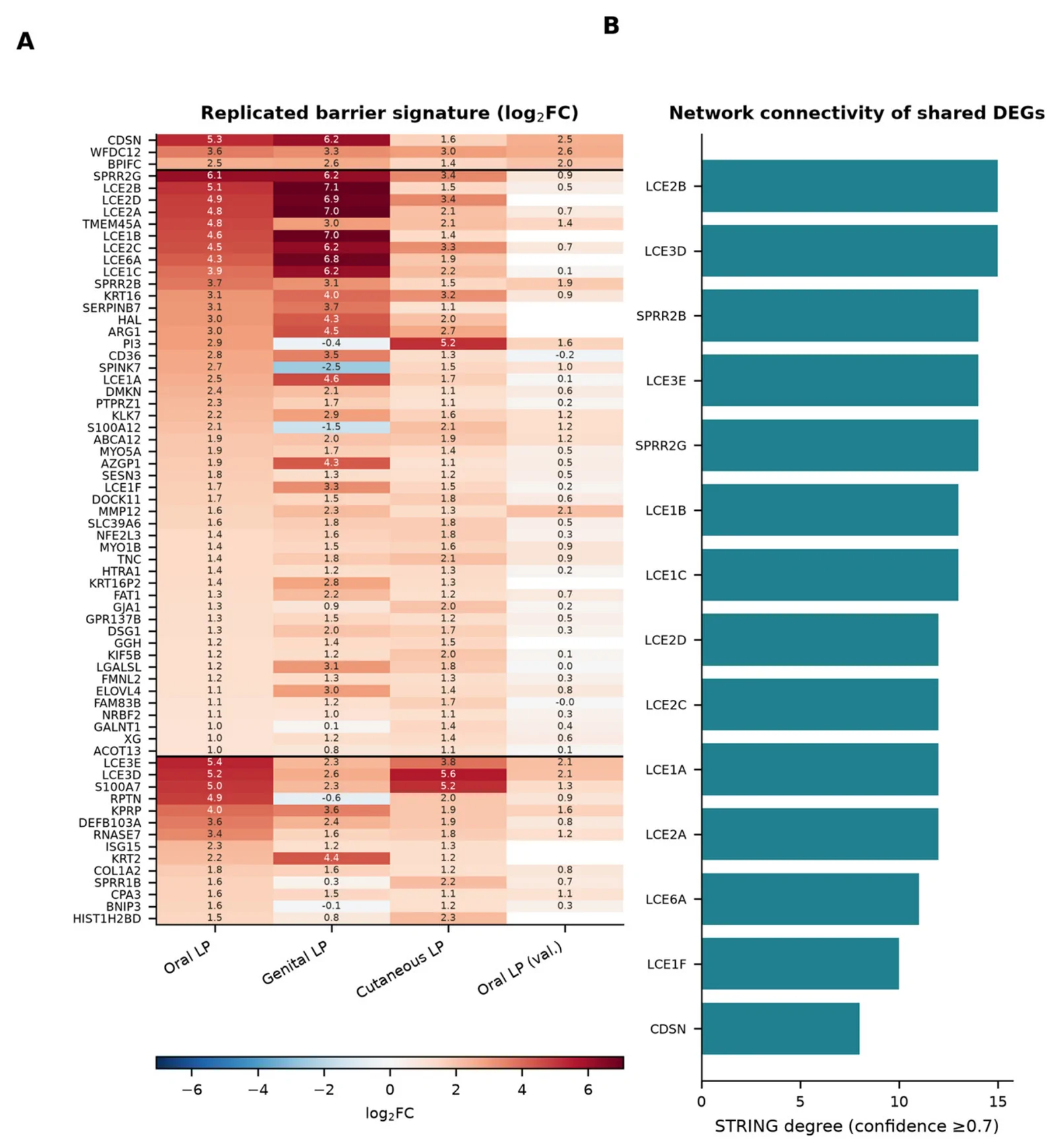
Replication-graded shared signature. Heatmap of log_2_ fold changes across the four cohorts for Tier 1–Tier 3 genes (**A**), ordered by evidence tier, with STRING degree centrality shown alongside (**B**).

Eighty-five genes were differentially expressed in both oral and cutaneous LP; 83 of these changed in the same direction at both sites, and 2 (MT1G, MT1H) changed in opposite directions. The replicated signature therefore comprises 83 concordant genes within a shared set of 85. Grading the concordant genes by replication produced 3 Tier 1 genes significant in all four cohorts (BPIFC, CDSN, WFDC12), 49 Tier 2 genes significant in three cohorts (including DSG1, PI3, KRT16, KLK7, SPRR2B, SPRR2G, LCE1A–LCE2D, MMP12, S100A12, ARG1, HAL, SPINK7, and TNC), and 31 Tier 3 genes significant in two cohorts; the 2 discordant genes are reported separately and were excluded from the barrier score (Figure 3, Table S12). Over-representation analysis of the shared set was dominated by formation of the cornified envelope (21 of 129 genes, q = 1.6 × 10^−25^) and keratinisation (Table S15).

Leave-one-out analysis showed that the shared hubs were robust: nine of ten tested hub genes retained significance in 14 of 14 oral iterations and 50 of 50 cutaneous iterations, the single exception being SPRR2E, which was unstable in the cutaneous cohort (0 of 50) and is therefore excluded from the signature (Table S20). Of the Tier 1 and Tier 2 genes, 41 had documented protein expression in oral mucosa or skin in the Human Protein Atlas (Table S26).

### 2.4. Network Structure of the Shared Signature

**Figure 4:**
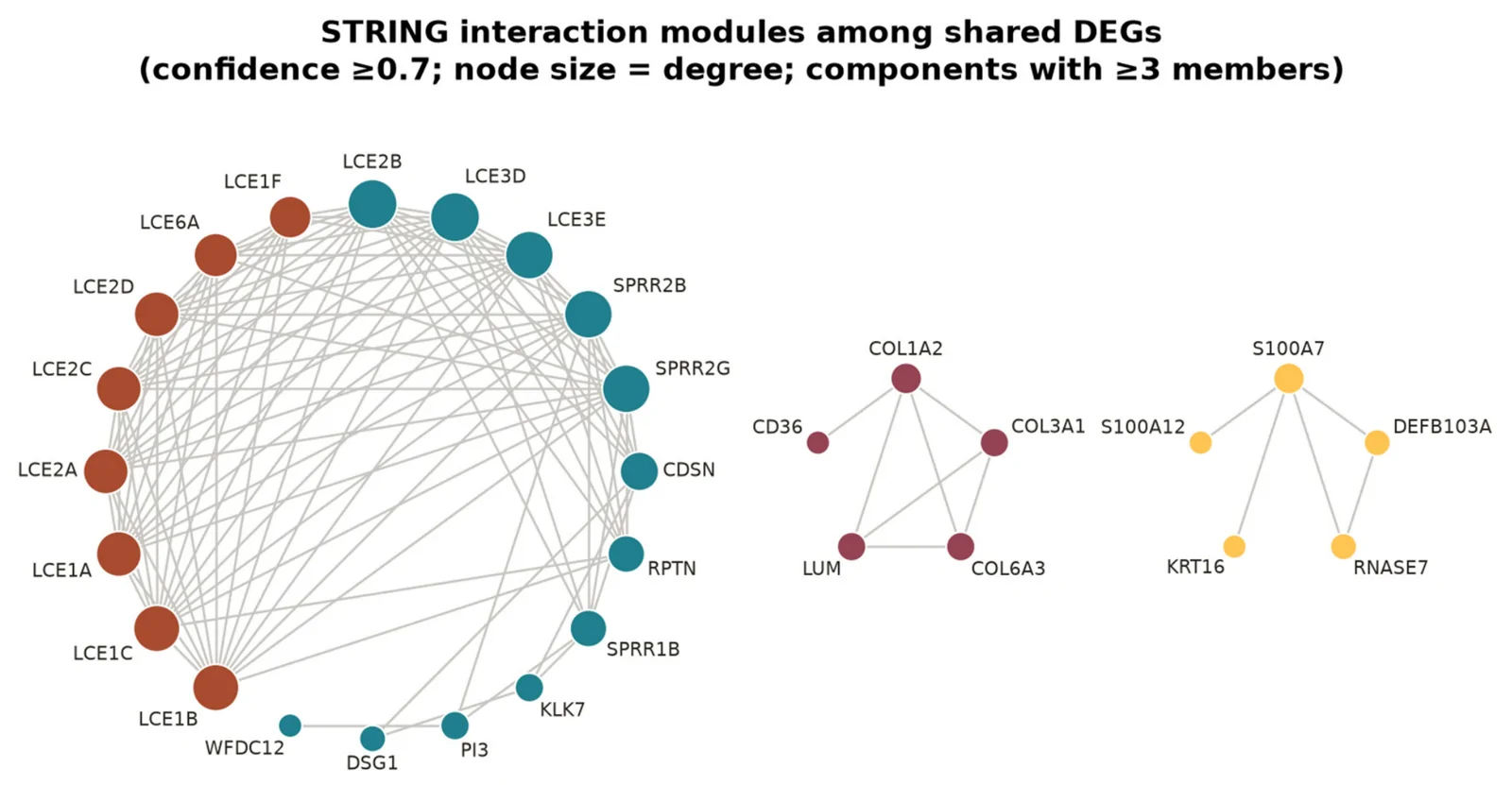
Protein–protein interaction structure of the shared signature. High-confidence STRING interactions (combined score ≥ 700) among the 85 shared genes. For legibility, only connected components with at least three members are drawn, so genes without high-confidence partners and isolated pairs are omitted; the full node and edge lists, including all isolated genes, are given in Tables S21 and S22. Node area is proportional to degree within the drawn network, and node colour denotes the greedy-modularity module: the dominant cornified-envelope module (**left**), a collagen/matrix module (**centre**) and an S100/defensin antimicrobial module (**right**). Nodes are arranged on a circle within each component, grouped by module, and edge geometry carries no information.

High-confidence STRING interactions among the shared genes produced a network of 85 nodes and 117 edges (density 0.033) organised into a dominant cornified-envelope module (LCE2B, LCE3D, SPRR2B, LCE3E, and SPRR2G as top-degree nodes), a smaller collagen/matrix module and an S100/defensin antimicrobial module (Figure 4, Tables S21 and S22). We stress that the topological prominence of individual LCE and SPRR paralogues is a property of a physically clustered, highly interconnected gene family and does not by itself identify any paralogue as a functional driver; this is precisely the inference that network-topology-only hub nomination cannot support.

### 2.5. Apparent Oral-Versus-Cutaneous Divergence Tracks Biopsy Compartment

**Figure 5:**
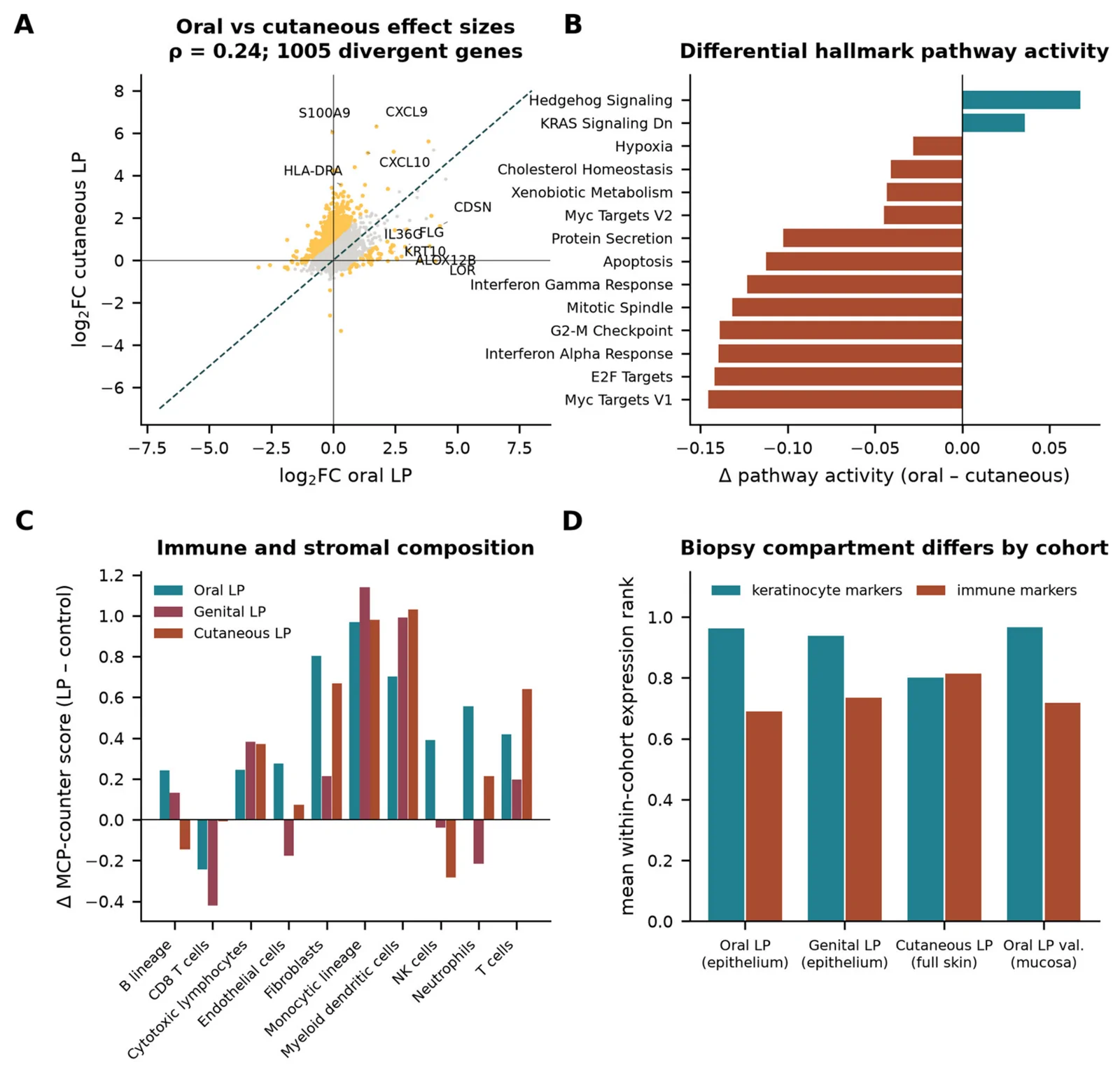
Site comparison and the compartment confounder. (**A**) Oral versus cutaneous effect sizes for all commonly tested genes, divergent genes highlighted. (**B**) Differential hallmark pathway activity (oral–cutaneous). (**C**) MCP-counter immune and stromal scores (lesional–control) by cohort. (**D**) Compartment proxy: mean within-cohort expression rank of keratinocyte and immune markers by cohort.

Across 6829 commonly tested genes, oral and cutaneous effect sizes correlated only weakly (Spearman ρ = 0.24 overall; ρ = 0.41 among genes significant in either cohort, 61.2% concordant in direction), and 3502 genes differed significantly between sites (1005 with an effect-size difference ≥ 1; Figure 5A, Table S13). Genes with larger oral effects were cornification genes (LOR, KRT17, ALOX12B, CDSN, IL36G, ALOXE3, FLG, and KRT10), whereas genes with larger cutaneous effects were interferon-inducible and myeloid (S100A9, CXCL9, S100A8, CXCL10, HLA-DRA, DSG3). Hallmark activity mirrored this: interferon-α (Δ = 0.198), interferon-γ (Δ = 0.178), complement, and MYC-target activity were higher in cutaneous LP, and only hedgehog and KRAS-down programmes were higher in oral LP (Figure 5B, Tables S16 and S17). MCP-counter estimates showed significant increases in monocytic lineage, myeloid dendritic cells, T cells, fibroblasts, neutrophils, and cytotoxic lymphocytes in cutaneous LP only (Figure 5C, Table S18).

The compartment proxy explains this pattern. Keratinocyte and immune marker ranks were 0.96 and 0.69 in oral LP, 0.94 and 0.74 in genital LP, the profile of separated epithelium, and 0.97 and 0.72 in the independent oral cohort, but 0.80 and 0.81 in the cutaneous cohort, the profile of full-thickness tissue (Figure 5D, Table S19). In other words, the GSE52130 mucosal arrays largely exclude the lichenoid infiltrate that the cutaneous arrays include. The interferon and immune-cell “divergence” between oral and cutaneous LP is therefore not interpretable as a difference in disease biology, and we do not interpret it as such. By contrast, the oral excess of cornification signal is measured in the compartment where those genes are expressed and is consistent with the within-study analysis below.

**Figure 6:**
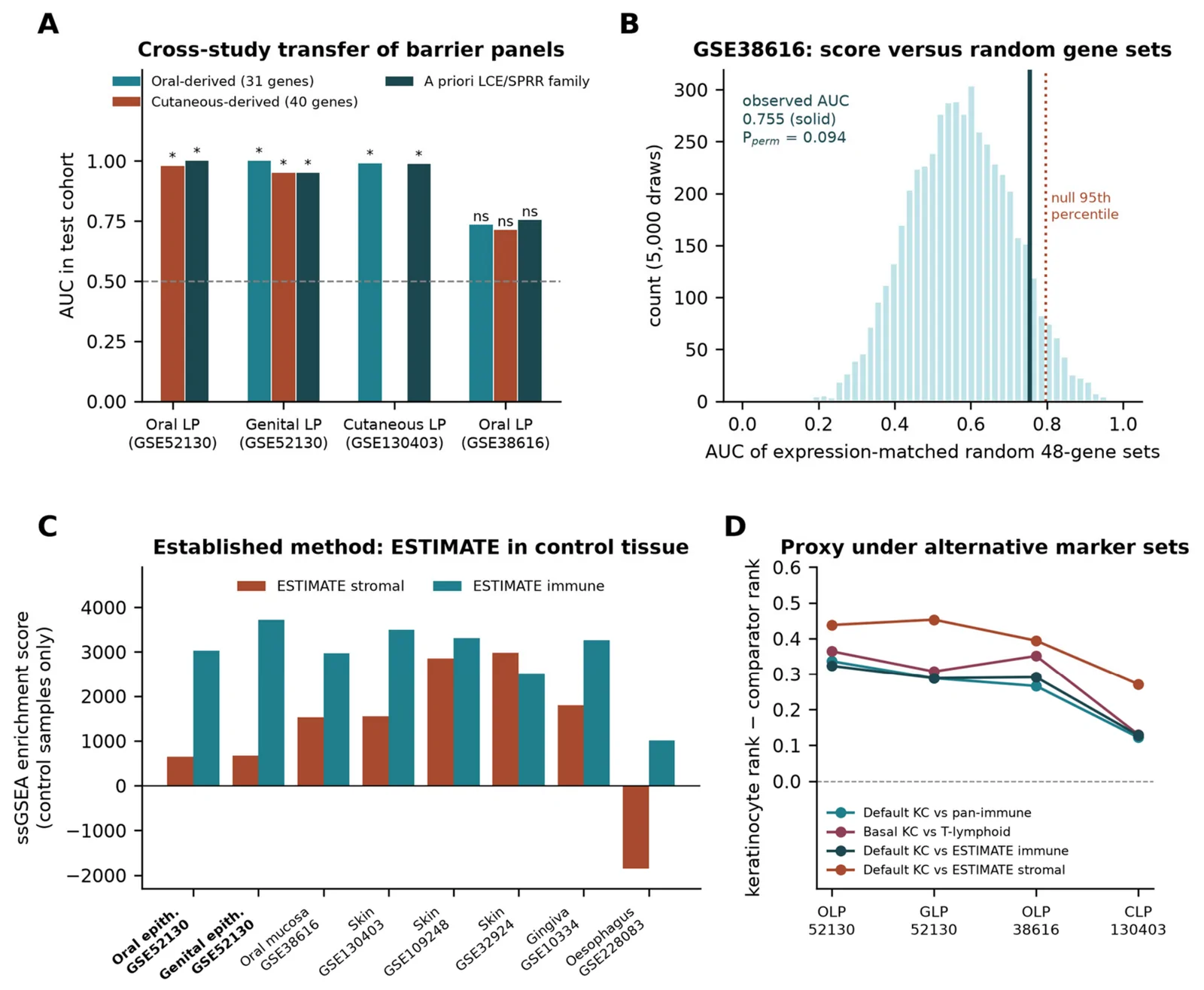
Cross-study validation of the barrier score and sensitivity of the compartment proxy. (**A**) AUC in each test cohort of keratinisation panels derived from oral LP only (31 genes) or cutaneous LP only (40 genes), and of an a priori LCE/SPRR family score requiring no derivation; derivation cohorts are not tested on themselves; * *p* < 0.05, ns not significant (Mann–Whitney). (**B**) Null distribution of the AUC in GSE38616 for 5000 random gene sets matched to the 48 measurable panel genes by expression level, with the observed AUC (solid line) and the null 95th percentile (dotted line). (**C**) ESTIMATE stromal and immune ssGSEA scores in control samples of the LP and comparator series; epithelium-only series are labelled in bold. (**D**) Keratinocyte-minus-comparator rank gap in the four LP cohorts under four marker-set combinations.

Because this proxy is a custom metric, we tested its sensitivity to marker choice and compared it with an established method (Figure 6C,D; Table S36). Replacing the keratinocyte markers with basal and structural keratinocyte genes that are not part of the cornification response, and the immune markers with a T-lymphoid-only set, preserved the ordering: the keratinocyte-minus-immune rank gap was 0.31–0.36 in the three mucosal cohorts and 0.13 in cutaneous LP. Across 1000 random 70% subsamples of the marker sets, the 95% intervals of the gap were 0.26–0.41 (oral), 0.21–0.36 (genital), 0.19–0.33 (independent oral) and 0.04–0.22 (cutaneous). The ESTIMATE immune and stromal signatures [29], scored by ssGSEA in control samples, gave the clearest view of biopsy depth. Control GSE52130 oral and genital epithelium had the lowest stromal scores of eight tissues examined (644 and 671), compared with 1545 in control skin from GSE130403 and 1792–2984 in full-thickness skin and gingiva from the comparator series. The lesional rise in ESTIMATE immune score was also larger in cutaneous LP (+2789) than in oral (+848) or genital (+971) LP epithelium. One qualification emerged. The independent oral cohort GSE38616, annotated only as oral mucosa, had a control stromal score (1522) comparable to skin, so it is better regarded as a mucosal biopsy containing lamina propria than as separated epithelium, and its lesional immune rise was intermediate (+1472). The compartment argument therefore rests mainly on GSE52130.

### 2.6. Within-Study Divergence Between Two Mucosal Sites

**Figure 7:**
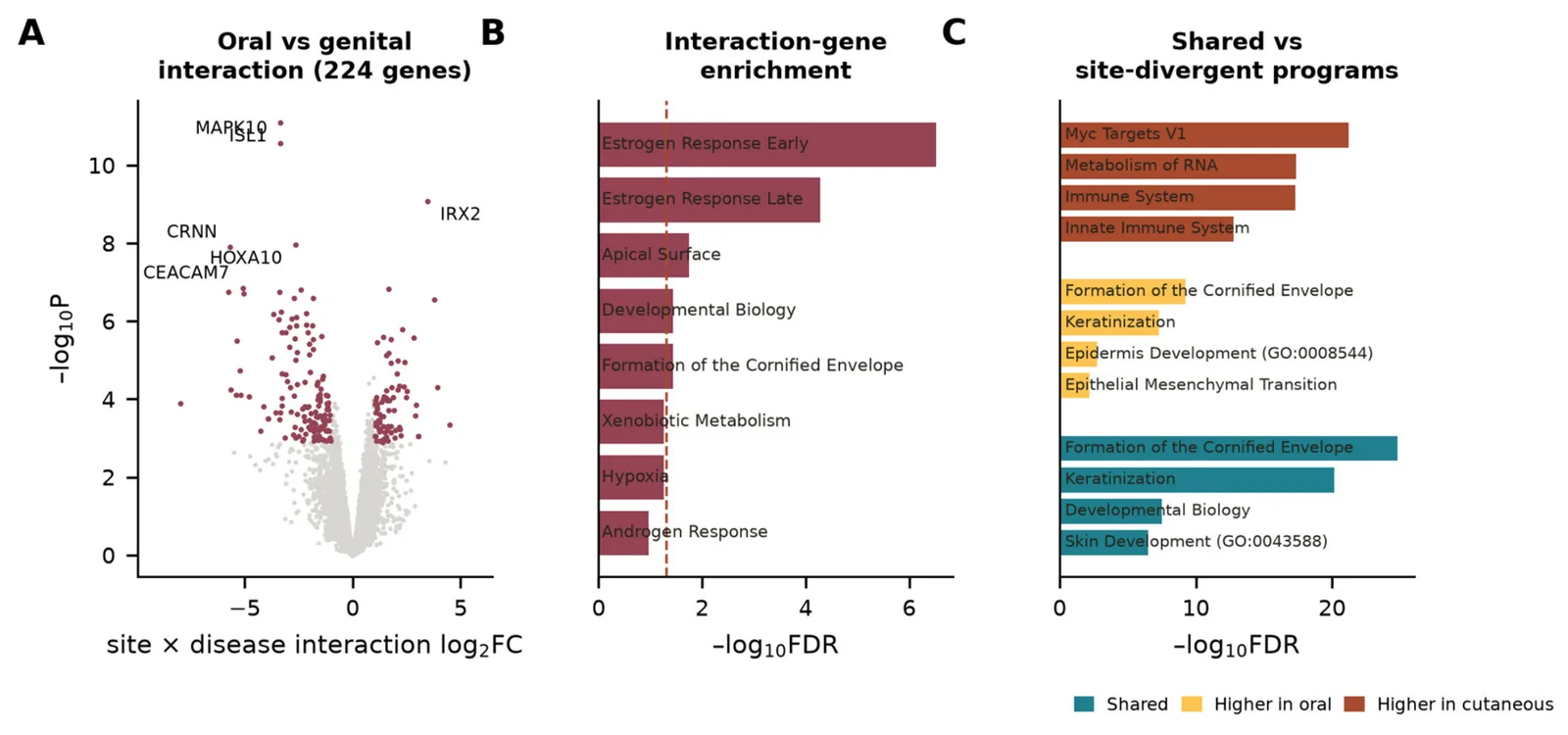
Within-study site specificity. (**A**) Volcano plot of the disease × site (oral versus genital) interaction fitted within GSE52130; (**B**) over-representation results for the interaction genes; and (**C**) comparison of shared and site-divergent programmes between oral and cutaneous LP.

The interaction model fitted within GSE52130 alone identified 292 genes with a significant disease × site (oral versus genital) interaction at FDR < 0.05, of which 224 had |log_2_ fold change| ≥ 1 (top genes ISL1, MAPK10, IRX2, CRNN, HOXA10, CXCL17, and MAL; Figure 7, Table S14). These genes were enriched for oestrogen response (early and late) and for cornified-envelope programmes (Table S15). Overall, the two mucosal sites were highly concordant (ρ = 0.74 among significant genes; 85.8% in the same direction), so site divergence within mucosa is a modulation of a common programme, concentrated in hormone-responsive and regional-keratinisation genes, rather than a different disease. This is biologically coherent: regional keratinisation patterns in oral mucosa are spatially organised [30,31,32], and the vulvovaginal epithelium is under direct steroid-hormone control [33,34,35,36].

### 2.7. A Barrier Score Separates Lesional from Control Tissue Within and Between Discovery Studies

**Figure 8:**
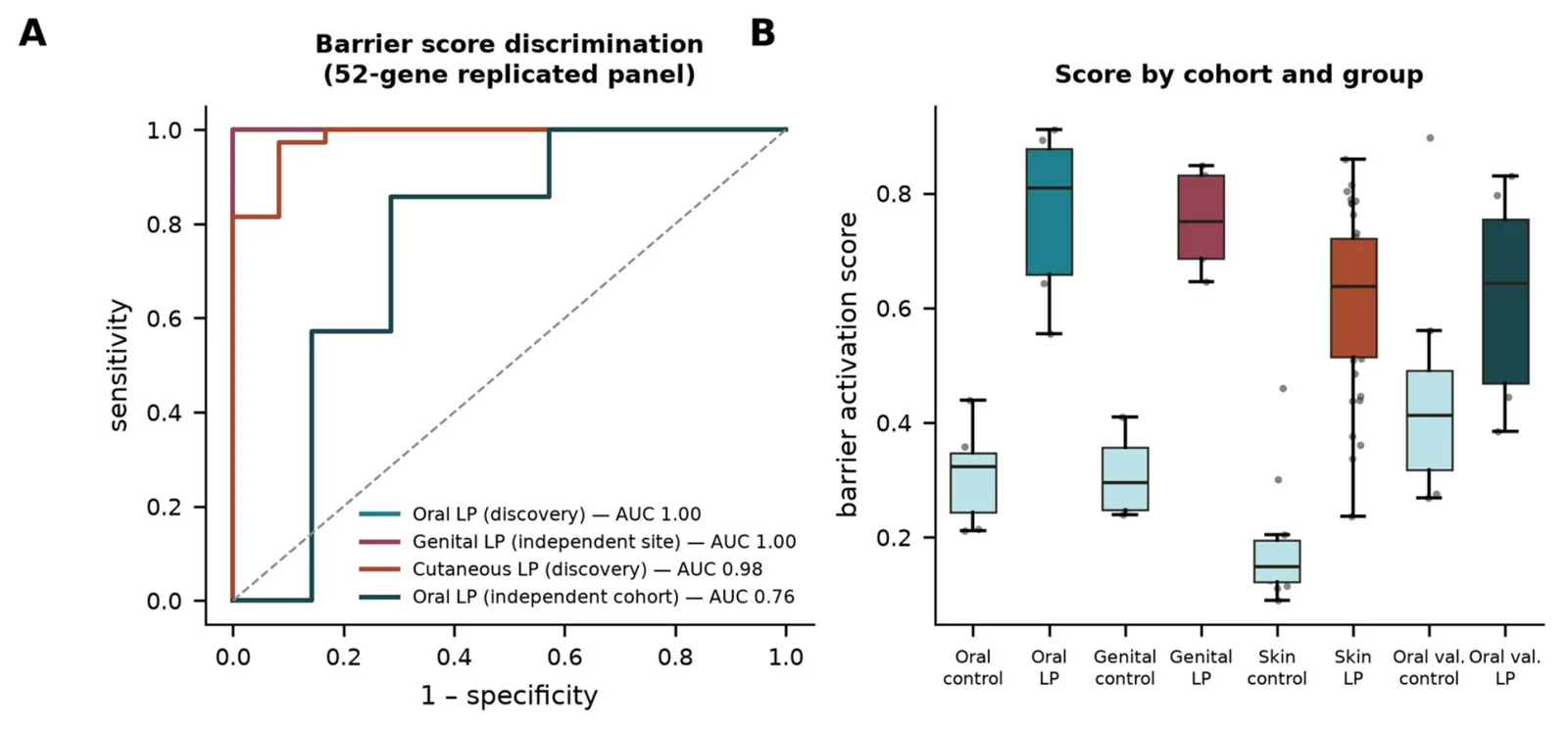
Barrier score performance. (**A**) Receiver-operating-characteristic curves for the 52-gene barrier score in each cohort with AUC values, and (**B**) per-sample score distributions by group.

The 52-gene barrier score separated lesional from control samples with AUC 1.00 in the oral discovery arm (*p* = 5.8 × 10^−4^), 1.00 in the genital arm (*p* = 0.016), 0.982 in cutaneous skin (*p* = 6.2 × 10^−7^) and 0.755 in the independent oral cohort (*p* = 0.128; Figure 8, Table S25). These estimates differ in what they can support. The oral and cutaneous values are in-sample: both cohorts were used to derive the panel, so their AUCs describe internal coherence rather than generalisation. The genital arm was not used for derivation and provides an out-of-derivation estimate, although it shares a study and platform with the oral discovery arm. The independent oral cohort GSE38616 is the only fully independent test, and there the score showed an AUC above 0.5 but without statistical significance in 7 versus 7 samples. Generalisation is therefore supported within GSE52130 and only suggestive across studies. The score is presented as a reproducibility metric for the shared programme, not as a diagnostic test; LP diagnosis is clinicopathological, and no diagnostic claim is made.

To obtain a non-circular test of cross-study transfer, we re-derived barrier panels from one discovery cohort only, restricting candidates a priori to Reactome keratinisation and cornified-envelope genes, and scored them in cohorts never used for their derivation (Figure 6A, Table S35). A 40-gene panel derived from cutaneous LP alone separated oral (AUC 0.98, *p* = 0.001) and genital (AUC 0.95, *p* = 0.032) LP epithelium in GSE52130, which differs in study, platform, laboratory, compartment, and anatomical site. A 31-gene panel derived from oral LP alone separated cutaneous LP (AUC 0.99, *p* = 3.8 × 10^−7^) and genital LP (AUC 1.00, *p* = 0.016). A score built with no derivation step, from all measured LCE and SPRR family genes, gave AUC 1.00, 0.95 and 0.99 in the same three cohorts. In GSE38616, by contrast, every panel gave AUC 0.71–0.76, and none reached significance. This independent-cohort result is directionally consistent but underpowered. With 7 versus 7 samples, the smallest AUC that can reach two-sided *p* < 0.05 is 0.84; the bootstrap 95% confidence interval of the observed AUC was 0.45–1.00; and the score exceeded 90.6% of 5000 random gene sets matched for expression level (permutation *p* = 0.094; null median 0.57, 95th percentile 0.80; Figure 6B). Of 76 measurable signature genes, 69 changed in the same direction as in discovery (binomial *p* = 6.4 × 10^−14^), and 10 were nominally significant in the concordant direction (*p* = 2 × 10^−5^), but the fold changes were about three-fold smaller (median |log_2_FC| 0.57 versus 1.65). We therefore regard transfer between the two discovery studies as established, and transfer to GSE38616 as not demonstrated.

### 2.8. MicroRNAs Previously Nominated by Network Topology Are Not Supported by Tissue Data

**Figure 9:**
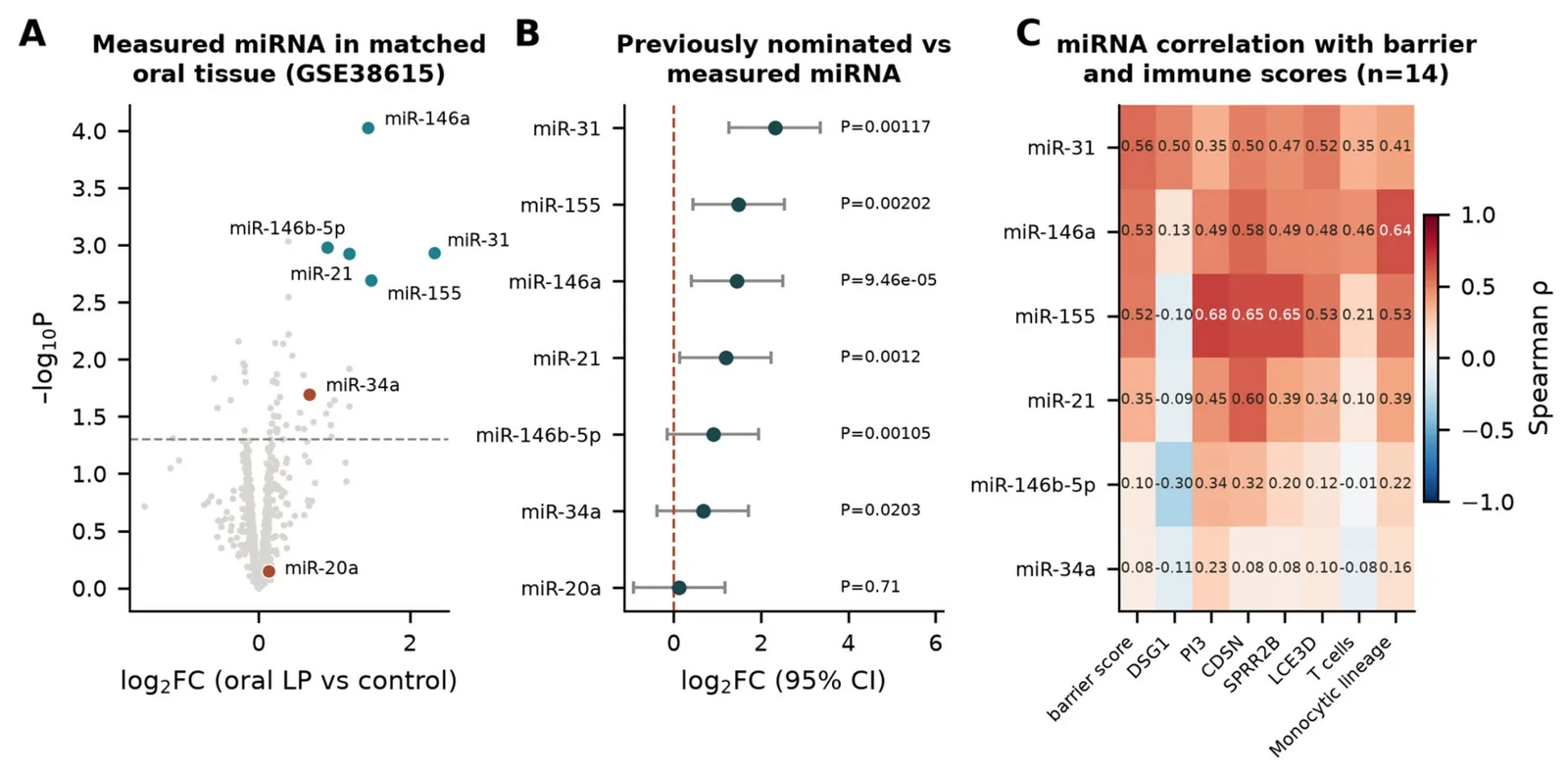
MicroRNA analysis in matched oral tissue (GSE38615, 14 biopsies). (**A**) Volcano plot of microRNA differential expression between lesional and control oral tissue; previously nominated candidates (miR-20a-5p, miR-34a-5p) are shown in rust and microRNAs reaching nominal significance in teal, with the dashed line marking *p* = 0.05. (**B**) Differential-expression effect estimates for the previously nominated and the most strongly changed microRNAs, shown as log_2_ fold change with 95% confidence intervals and nominal *p* values; the dashed vertical line marks no change. (**C**) Spearman correlation coefficients between microRNA expression and the barrier score, individual barrier genes (DSG1, PI3, CDSN, SPRR2B, and LCE3D) and MCP-counter immune estimates (T cells and monocytic lineage) across the same 14 biopsies; positive correlations with predicted targets do not support the expected inverse relationship under simple canonical microRNA-mediated repression. In (**A**), all other microRNAs are shown in grey; in (**C**), cell values give Spearman ρ, with red indicating positive and blue negative correlation (see colour bar).

In the matched microRNA arrays, 52 microRNAs were nominally differentially expressed in OLP (none survived FDR correction in the axis analysis; Table S23). Neither of the microRNAs nominated in earlier network-based work was associated with the shared barrier programme: miR-20a-5p versus barrier score ρ = 0.138 (*p* = 0.637) and miR-34a-5p ρ = 0.077 (*p* = 0.794). The strongest observed associations were unrelated to that hypothesis and were mostly positive rather than inverse (miR-548a-3p ρ = −0.653, miR-181b ρ = 0.618, miR-31 ρ = 0.565, miR-146a ρ = 0.525), and none survived FDR correction; a single target-level association, miR-155 versus PI3 (ρ = 0.684, *p* = 0.007), was also positive and therefore not consistent with canonical repression (Figure 9, Table S24). We report this as a negative result: with 14 matched biopsies, tissue microRNA data do not support a miR-20a-5p/miR-34a-5p axis regulating the shared barrier programme, and any microRNA nominated from database-predicted or network-derived topology alone should be treated as an untested hypothesis [37,38].

### 2.9. Experimentally Supported microRNA–Target Pairs Show Positive, Not Inverse, Coupling with the Barrier Programme

**Figure 10:**
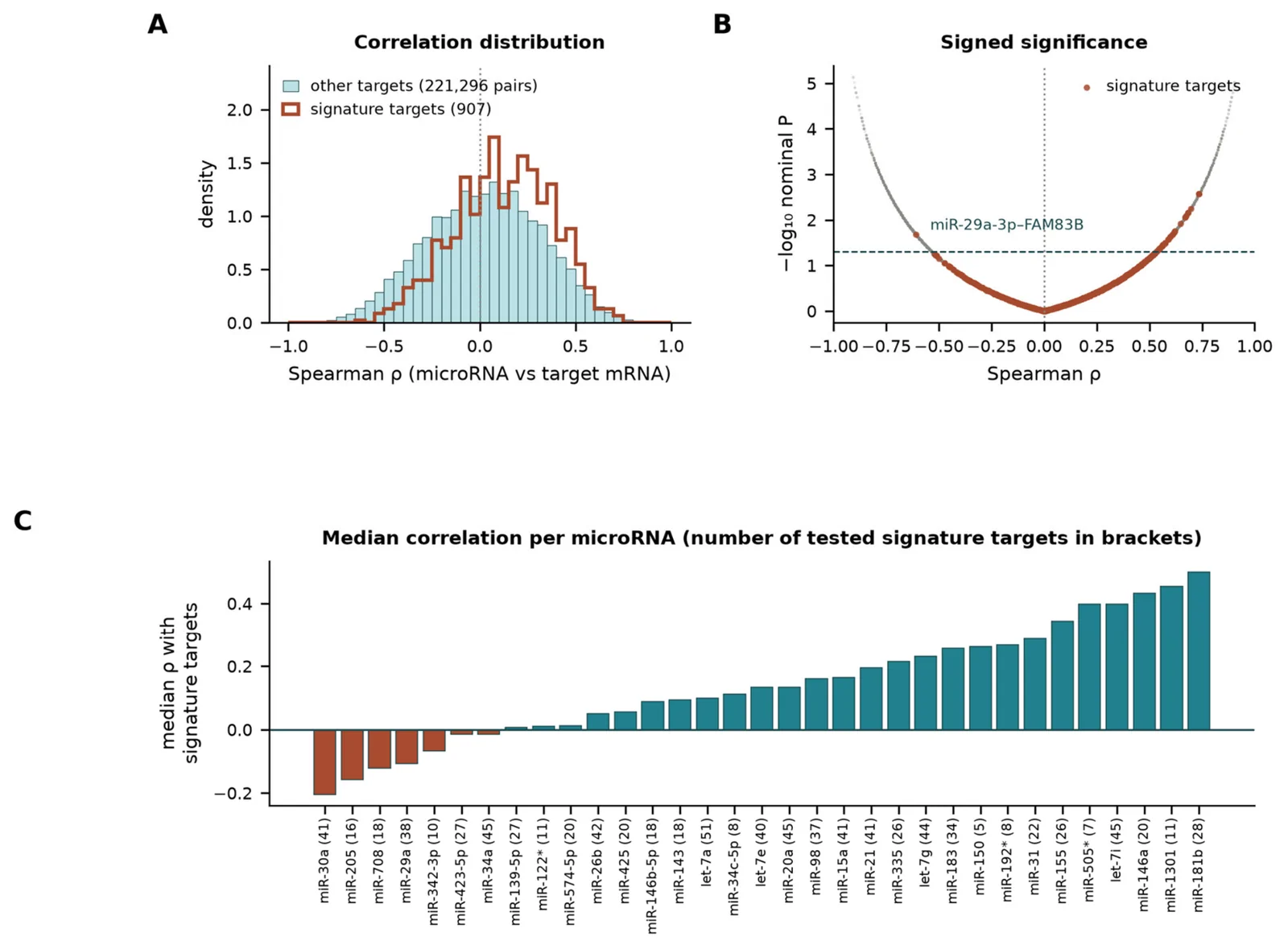
Experimentally supported microRNA–target associations in the same 14 matched biopsies (GSE38615 microRNA and GSE38616 mRNA arrays). (**A**) Distribution of Spearman correlation coefficients for the 221,296 testable microRNA–target pairs outside the shared signature (filled histogram) and for the 907 pairs whose target belongs to the 83-gene concordant signature (outlined histogram); dotted vertical lines mark zero correlation, and the signature distribution is displaced towards positive rather than negative values. (**B**) Signed significance of every tested pair, plotted as correlation coefficient against −log_10_ nominal *p* value, with signature pairs highlighted and all other pairs in grey; the horizontal dashed line marks nominal *p* = 0.05 and the single nominally inverse signature pair (miR-29a-3p–FAM83B) is labelled. No pair passed false-discovery-rate correction. (**C**) Median correlation coefficient with conserved signature targets for each microRNA, ordered by value, with the number of tested signature pairs in brackets; microRNAs with a negative median are shown in rust and those with a positive median in teal. Counts in panel C are per array probe and therefore combine both mature forms where a probe maps to more than one, so they exceed the mature-form counts quoted in the text. Asterisks (miR-122*, miR-192*, miR-505*) denote the minor strand under the legacy miRBase nomenclature used by the array annotation (current names: miR-122-3p, miR-192-3p and miR-505-5p).

To test the cross-site regulatory hypothesis on validated rather than predicted interactions, 286,846 experimentally supported interaction records were retrieved for the 53 microRNAs carried forward; 51 had at least one supported target measured on the matched mRNA arrays, giving 222,203 testable pairs across 15,704 genes. The resulting correlation distribution was not shifted towards repression: 46.3% of pairs were negative, significantly fewer than the 50% expected by chance (binomial *p* = 4.5 × 10^−260^), with median ρ = +0.029, and 6636 pairs reached nominal inverse significance against 8149 nominally positive pairs and approximately 5555 expected under the null. No pair survived FDR correction (minimum q = 0.21), including the strongest inverse candidates (miR-26b–LANCL2, ρ = −0.91, *p* = 7 × 10^−6^; Figure 10, Table S28).

Within the 83-gene concordant signature, the result ran opposite to the hypothesis. Fifty-seven signature genes carried at least one supported interaction, yielding 907 pairs, of which only 31.0% were negative (binomial *p* = 6.1 × 10^−31^), with median ρ = +0.138, significantly higher than non-signature pairs (Mann–Whitney *p* = 2.1 × 10^−26^). Thirty-nine pairs were nominally positive, and one was nominally inverse (miR-29a-3p–FAM83B, ρ = −0.609, *p* = 0.021, q = 0.67; TarBase record of negative regulation, PMID 23820384), and none survived FDR correction (minimum q = 0.49). The previously nominated candidates behaved the same way at target level: miR-20a-5p had 40 supported pairs within the signature, and miR-34a-5p had 44, with no nominally inverse pair in either case (median ρ = +0.112 and −0.020). The 11 anti-correlated microRNA–mRNA pairs originally reported from these same biopsies [39] lie almost entirely outside the experimentally supported interaction space: only two (miR-15a–MRPS26, miR-21–SNTG1) are recorded in TarBase or miRTarBase, of which miR-21–SNTG1 reproduced as an inverse correlation here (ρ = −0.53, *p* = 0.049), and none involves a gene in the conserved barrier signature (Figure 10, Table S29).

Independent tissue evidence for the microRNAs implicated by this test is also asymmetric between sites, which limits how far any axis can be extended across the LP spectrum. Of the microRNAs changed in this oral cohort, only miR-155-5p and miR-203a-3p have been measured in cutaneous LP lesional skin at all: miR-155 is higher in classic cutaneous LP than in control skin [40], and miR-203 and miR-125b are lower [41], whereas miR-203 is reported as higher in oral LP epithelium [42], so its reported direction inverts between compartments. miR-29a-3p, the only microRNA contributing a nominally inverse signature pair here, has independently been found elevated in OLP biopsy tissue [37] and has a luciferase-validated keratinocyte adhesion target, desmocollin-2 [43], but has never been measured in cutaneous LP. The validated epidermal-differentiation targets of the other changed microRNAs, keratins 16 and 17 for miR-31 [44] and loricrin for miR-155 [45], are keratin and envelope genes lying outside the LCE/SPRR core of our signature (Table S30).

### 2.10. Specificity of the Shared Barrier Programme Against Other Inflammatory Epithelial Diseases

**Figure 11:**
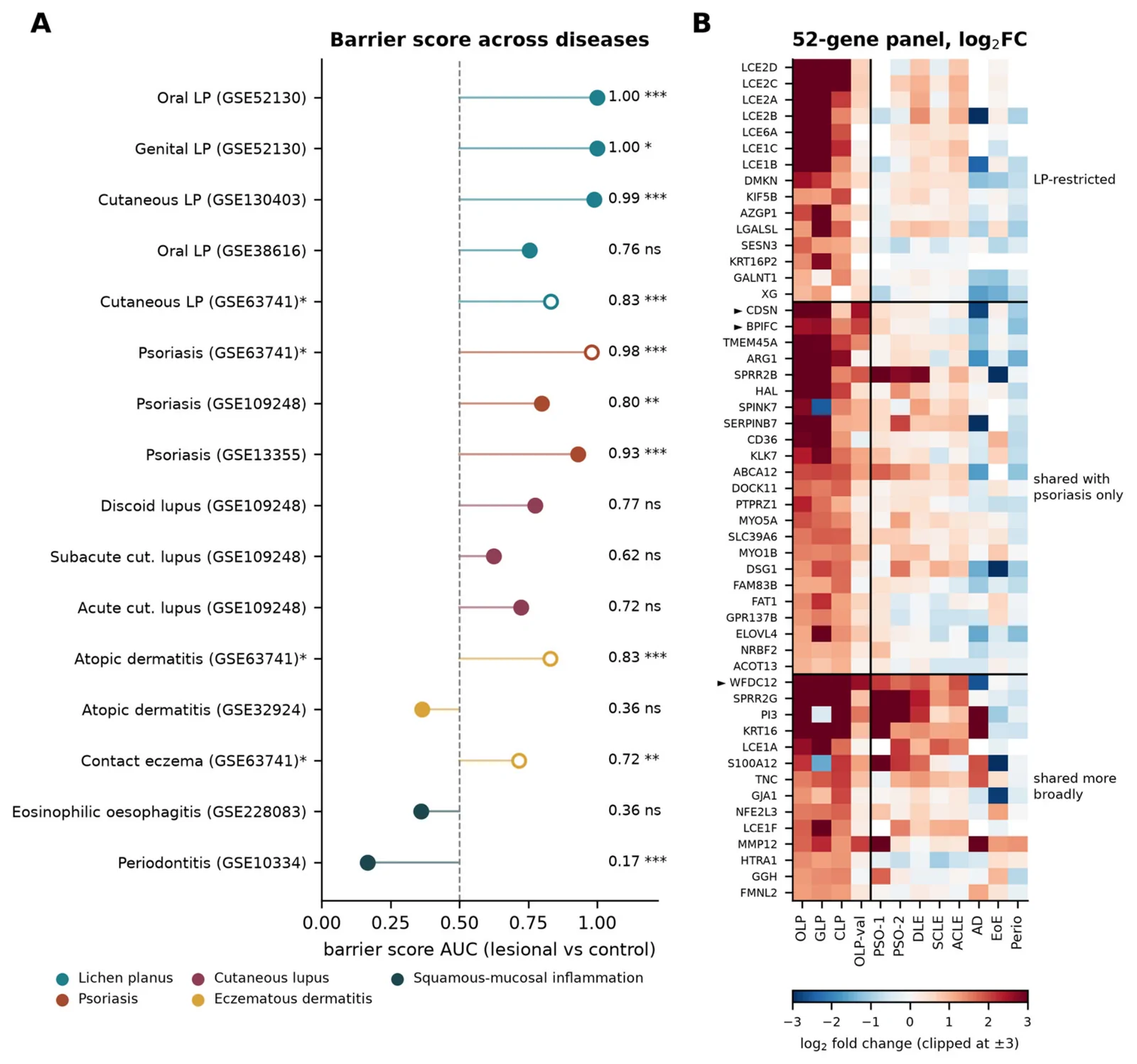
Specificity of the shared barrier programme. (**A**) AUC of the unchanged 52-gene barrier score for lesional versus control tissue in the LP cohorts and in comparator series of other inflammatory skin and mucosal diseases; filled symbols, at least 40 panel genes measured; open symbols and asterisked labels, GSE63741 skin-focused array with 12 panel genes; *** *p* < 0.001, ** *p* < 0.01, * *p* < 0.05, ns not significant (Mann–Whitney). (**B**) Log_2_ fold changes (lesional versus control) of the 52 panel genes, grouped by whether each gene is concordantly changed (FDR < 0.05) in no comparator, only in psoriasis, or in at least one non-psoriatic comparator; Tier 1 genes are marked with arrowheads. OLP, oral LP (GSE52130); GLP, genital LP (GSE52130); CLP, cutaneous LP (GSE130403); OLP-val, oral LP (GSE38616); PSO-1, psoriasis (GSE13355); PSO-2, psoriasis (GSE109248); DLE, SCLE and ACLE, discoid, subacute and acute cutaneous lupus (GSE109248); AD, atopic dermatitis (GSE32924); EoE, eosinophilic oesophagitis (GSE228083); Perio, periodontitis (GSE10334).

To ask whether the shared programme is specific to LP or reflects a more general response of inflamed epithelium, we applied the 52-gene barrier score and the 83-gene concordant signature, unchanged, to six public series covering psoriasis (three series), discoid, subacute and acute cutaneous lupus, atopic dermatitis, contact eczema, eosinophilic oesophagitis and periodontitis, including one series in which LP and other dermatoses were profiled together (Table S31) [26,46,47,48,49,50]. The programme was not specific to LP (Figure 11A, Table S32). It was strongly induced in psoriasis, with AUC 0.93 (*p* = 2.3 × 10^−16^) in GSE13355 and 0.80 (*p* = 0.005) in GSE109248, which uses the same Illumina platform as GSE52130. It was partly induced in cutaneous lupus (AUC 0.77, 0.62 and 0.72 for discoid, subacute and acute disease; none significant). It was not induced in lesional atopic dermatitis (AUC 0.36) or eosinophilic oesophagitis (AUC 0.36), and it was reversed in periodontitis (AUC 0.17, *p* = 1.8 × 10^−15^), in which most barrier genes were lower in diseased gingiva. In GSE63741, where only 12 panel genes are present on the skin-focused array, the score separated LP from healthy skin (AUC 0.90) but not from atopic dermatitis (AUC 0.52 for LP versus atopic dermatitis) or contact eczema (0.68), and it was higher in psoriasis than in LP (AUC 0.22 for LP versus psoriasis).

At gene level (Figure 11B, Table S33), the proportion of the 83 signature genes changed concordantly at FDR < 0.05 was 54–99% in the oral, genital and cutaneous LP cohorts (0% in the underpowered GSE38616), 33–65% in psoriasis, 5–13% in cutaneous lupus, 9% in atopic dermatitis, 5% in eosinophilic oesophagitis and 14% in periodontitis. Of the 52 panel genes, 14 were concordantly changed in at least one non-psoriatic comparator, 23 were shared with psoriasis only, and 15 were not changed in any comparator. All three Tier 1 genes were shared with psoriasis. BPIFC and CDSN were induced in psoriasis only (GSE13355 log_2_FC 0.66 and 0.50), whereas WFDC12 was induced in all three psoriasis series and in discoid and acute cutaneous lupus (log_2_FC 1.94 and 1.91). The 15 genes not changed in any comparator were dominated by the LCE1/LCE2 cluster (LCE1B, LCE1C, LCE2A–LCE2D, and LCE6A). Consistent with this, an LCE1/LCE2 group score shifted more in the three LP discovery and genital cohorts (standardised difference 2.1–3.3) than in any comparator (≤1.4). Psoriasis, by contrast, was dominated by LCE3 induction (standardised difference 4.1 for LCE3 versus 0.9 for LCE1/LCE2 in GSE109248; Table S34), in keeping with the LCE3 bias of psoriatic epidermis [27]. The LCE1/LCE2 feature was not reproduced in GSE38616 (standardised difference 0.2), so we treat it as a candidate distinguishing feature rather than an established one.

## 3. Discussion

Reanalysing every eligible public LP expression series in full, rather than the subsets used previously, produces three findings with different levels of confidence. The best-supported is that a cornified-envelope and keratinisation programme is dysregulated in LP at three anatomical sites, replicates across four cohorts and two integration strategies, survives leave-one-out and down-sampling perturbation, and transfers between the two discovery studies, although not significantly to the small independent oral cohort; it is, however, shared with psoriasis and partly with cutaneous lupus rather than specific to LP. The second is that the widely assumed immune-transcriptomic divergence between oral and cutaneous LP cannot be extracted from these data, because the mucosal and cutaneous series sampled different tissue compartments; our compartment proxy quantifies that confounder directly. The third, more limited but internally clean, is that two mucosal sites profiled on one platform diverge in a specific and interpretable way, through hormone-response and regional-keratinisation genes.

The shared programme is consistent with a growing body of work in which barrier genes are active participants in lichenoid inflammation rather than downstream markers of it. LCE3D links barrier proteins to TGF-β1 signalling in atopic dermatitis [18], SPRR2B knockdown suppresses keratinocyte proliferation and inflammatory output [19], an SPRR2A–CSTA axis drives IL-17A-induced squamous metaplasia and steroid resistance [20], elafin (PI3) is a bona fide inflammatory effector in psoriatic epithelium [51], and dysregulated corneodesmosome proteolysis produces severe barrier disease when CDSN and its regulators fail [52,53]. Comparative transcriptomics of the epidermal differentiation complex shows the same dual structural/immunological role [17]. Our data place LP in this framework at three sites simultaneously and identify BPIFC, CDSN, and WFDC12 as the only genes replicating in every cohort tested, a much narrower and more defensible claim than a topology-derived hub list.

The three genes that replicated in every LP cohort have coherent but distinct barrier roles. CDSN encodes corneodesmosin, an adhesive glycoprotein of corneodesmosomes at the transition from granular to cornified layer. Its complete loss causes peeling skin disease with a severe barrier defect, pruritus and atopy [54]; dominant truncating variants cause hypotrichosis simplex of the scalp [55]; and its deletion in mouse epidermis produces lethal barrier failure [56,57]. WFDC12 is a whey acidic protein four-disulfide-core protease inhibitor expressed specifically in terminally differentiated keratinocytes, where it localises to corneodesmosomes and inhibits kallikrein-related peptidase 7 and neutrophil elastase [58]. Keratinocyte-restricted overexpression of WFDC12 in mice aggravates imiquimod-induced psoriasiform inflammation through dendritic-cell activation and Th1 responses [59], and atopic-like dermatitis through ALOX12/15 metabolism and platelet-activating factor [60]. BPIFC is the least characterised. It is a BPI-fold lipid- and lipopolysaccharide-binding protein first described with expression in skin [61], identified among genes implicated in epidermal barrier function [62], with RNA expression enriched in skin and tongue [63], and a heterozygous variant has been reported in hereditary trichilemmal cysts [64]. Together with KLK7, SPINK7 and SERPINB7 in Tier 2, CDSN and WFDC12 point to a corneodesmosome and desquamation module, a protease–antiprotease system controlling cohesion and shedding of the cornified layer, rather than to the cornified envelope alone. This fits the hyperkeratosis and hypergranulosis that define LP histologically, including in oral mucosa that is normally only partly keratinised [1,2].

The comparator analysis also limits how these genes can be used. All three are shared with psoriasis, and WFDC12 also with cutaneous lupus, so none is likely to discriminate LP from other keratinising inflammatory dermatoses, and the barrier score should not be developed as a diagnostic marker of LP. The genes may still serve as quantitative readouts of epithelial activation, for example, of treatment response, but this has not been tested. Whether they are drivers cannot be answered from cross-sectional bulk data, and the functional evidence is asymmetric. WFDC12 gain of function amplifies cutaneous inflammation in vivo [59,60]. For CDSN, the disease-causing direction is loss of function [54,56,57], so its up-regulation in LP is more naturally read as a compensatory or reactive response to accelerated epithelial turnover. For BPIFC, no functional data exist in epithelium. Knockdown and overexpression of these genes in organotypic oral and epidermal models exposed to LP-relevant cytokines [65] would distinguish these possibilities.

These results answer the specificity question directly. The shared LP programme is not LP-specific. It is a keratinising-epithelium injury response that LP shares most closely with psoriasis and, to a lesser degree, with the interface dermatitis of cutaneous lupus. It is absent from type 2-dominated atopic dermatitis and eosinophilic oesophagitis and reversed in periodontitis, in which the inflamed pocket epithelium is ulcerated rather than hyperkeratotic. The programme is therefore not a generic consequence of chronic epithelial inflammation, but neither is it a molecular signature of LP. What is distinctive in our data is its reproducibility across oral, genital and cutaneous sites within one disease, together with a candidate LCE1/LCE2-biased composition that requires independent confirmation. This agrees with earlier skin-focused profiling, in which LP was separated from psoriasis and atopic dermatitis by its CXCL9-dominated interferon profile rather than by barrier genes [28]. Two cautions apply. The comparators differ from the LP series in platform and laboratory, as the LP series do from one another, and the absence of induction in oesophageal and gingival disease may partly reflect tissue type as well as disease biology.

Our compartment finding is a methodological caution with immediate consequences for how public LP data are compared. Cellular composition dominates bulk transcriptomic contrasts in inflammatory skin disease [21,22], deconvolution estimates are highly sensitive to reference construction and to unrepresented cell types [23,24,25,66], and confounding by composition is now treated as a first-order problem in transcriptomic inference [67,68]. Studies that compare epithelium-only mucosal series with full-thickness cutaneous series will recover interferon and myeloid signal preferentially in skin, whatever the underlying biology. Where site comparison is the question, either the compartment must be matched by design, or the comparison must be made within a study, as in our interaction model. The methodological precedent for this style of batch-aware multi-cohort reanalysis in lichenoid disease is established [69], and single-cell and spatial studies, which resolve compartment explicitly, remain the appropriate route to cell-level cross-site comparison [9,11,15,70].

Platform heterogeneity is a second, partly separable source of between-cohort difference. The four LP series were generated on three array generations (Illumina HumanHT-12 v4 and Affymetrix HuGene 1.0 ST and 2.1 ST), with different probe content, normalisation, and RNA sources: GSE130403 was profiled from formalin-fixed, paraffin-embedded biopsies [8], whereas the mucosal series used fresh tissue. Probe content alone removed one to four panel genes outside GSE52130. Effect sizes were also compressed away from the discovery data. The median absolute log_2_ fold change of signature genes was 1.65–1.71 in GSE52130 and GSE130403, but 0.57 in GSE38616, and the slope of GSE38616 on GSE52130 fold changes was 0.22, although direction was preserved for 91% of genes (Table S37). Platform-dependent differences in fold-change magnitude with preserved direction are well documented in cross-platform comparisons [71,72]. Our analyses were designed to be robust to this: scores use within-cohort z-scores, discrimination is rank-based, replication is judged by direction and within-cohort significance, and the two oral cohorts were combined by both random-effects meta-analysis and ComBat with consistent results. Platform cannot, however, be fully separated from study, laboratory and compartment, because each is confounded with the others in the available data. For the specificity analysis, psoriasis profiled on the same Illumina platform as GSE52130 (GSE109248) reproduced the programme, whereas lupus on that platform did so only partly, so the pattern of sharing cannot be attributed to platform alone.

Interpreting the mucosal interaction result requires similar restraint. The enrichment of oestrogen-response programmes among oral-versus-genital interaction genes is consistent with the hormone dependence of vulvovaginal epithelium [33,34,35,36] and with the distinct clinical course and outcome measurement of vulvovaginal LP [73,74,75], but our data cannot establish that hormonal signalling modifies LP pathogenesis; the interaction genes may equally reflect constitutive regional differences in epithelial identity that are unmasked when a shared inflammatory programme is superimposed. Both explanations predict testable differences in hormone-receptor-directed experiments, which have not been performed here.

Three claims made in earlier analyses of these datasets, including our own, are not supported and should be retired. First, the nomination of miR-20a-5p and miR-34a-5p as candidate therapeutic microRNAs rested on network topology and predicted interactions; in matched tissue microRNA data from the same biopsies, neither correlates with the barrier programme, and the microRNAs that do correlate do not show the inverse relationship expected under a simple canonical repression model. Extending the test to experimentally supported interactions makes the point quantitative: across 222,203 validated microRNA–target pairs measurable in these biopsies, and in the 907 pairs involving conserved signature genes, correlations were predominantly positive, and no pair survived FDR correction, so lesional microRNA and barrier-gene abundance covary in the direction expected from co-induction within an activated epithelium or from shared shifts in cell composition, not from target repression. The one microRNA whose changes here have independent replication in oral LP tissue and a validated keratinocyte adhesion target, miR-29a-3p [37,43], has never been measured in cutaneous LP, and the only microRNA measured in both compartments with a consistent direction, miR-155-5p [40], correlates positively with its barrier targets in these data; miR-203a-3p, reported down in cutaneous LP skin [41] and up in oral LP epithelium [42], illustrates why microRNA findings should not be pooled across compartments at all. Existing OLP microRNA evidence is dominated by salivary and circulating measurements with inconsistent tissue-level replication [37,76,77,78,79,80], and the specific oncological roles reported for miR-34a-5p in cutaneous squamous carcinoma [81,82] cannot be transferred to lesional LP epithelium. Second, a 7-versus-7 cohort with no genome-wide significant genes is not a validation cohort. Third, expression-based nomination of pharmacological classes, namely NF-κB inhibitors, retinoids, vitamin D analogues, PPAR agonists and microRNA therapeutics, does not follow from a differential expression list; the therapeutic evidence that is actually accumulating in LP concerns JAK inhibition, which was developed from mechanistic and clinical work, not from transcript lists [83,84,85].

Our findings bear on malignant transformation only indirectly, and we make no predictive claim. Transformation rates are low and heterogeneous (0.44% under strict criteria; 2.28%, 95% CI 1.49–3.20, in the highest-quality quartile; 1% in extraoral LP, concentrated in vulvar disease) [3,4,6], and prediction currently rests on clinicopathological criteria and immunohistochemical markers, not on bulk barrier signatures [86,87,88]. The relevance of a persistently disrupted barrier with sustained keratinocyte activation is that it is a plausible context for the field effects described in that literature [89,90,91,92], and this is a hypothesis for longitudinal study rather than a result.

### Limitations

This is a reanalysis of publicly archived microarray data and carries the limitations of that design. No new tissue was collected and no experimental validation, whether protein, functional or in vitro, was performed; Human Protein Atlas records confirm only that the signature genes are expressed as protein in the relevant tissues, not that they are altered in LP. Cohorts are small (7–21 lesional samples per group; 14 matched microRNA pairs), so absence of significance in the independent oral cohort and in the microRNA analysis reflects limited power as well as effect size. The microRNA–target test is correspondingly weak: with 14 biopsies, a single pair has roughly 80% power only for |ρ| above about 0.7, and FDR correction across more than 200,000 bulk-tissue pairs is stringent, so it establishes the absence of a dominant, tissue-level inverse signal rather than the absence of any individual regulatory interaction, and bulk tissue cannot separate microRNA and target expression occurring in different cell populations. The barrier score was tested outside its derivation data in the genital arm, which shares a study and platform with the oral discovery cohort, in a reciprocal leave-one-cohort-out analysis between the two discovery studies, and in one small independent oral cohort in which the AUC was above 0.5 but not statistically significant, so transfer of the score to new studies remains provisional. Specificity was assessed only against diseases with suitable public array data. The comparator series differ in platform and, for oesophagus and gingiva, in tissue; the cutaneous lupus subgroups are small (6–12 lesional samples); periodontitis sites from the same patient were not modelled as clustered; and the most relevant clinical mimics, including lichenoid drug eruption, graft-versus-host disease, lichen sclerosus and oral leukoplakia, could not be tested. All series are bulk microarrays: transcript coverage is limited to array content, isoform and non-coding information is largely absent, and no cell-type-resolved inference is possible; where composition mattered, we quantified the confounder rather than removing it, since reference-based deconvolution of epithelium-only tissue is itself unreliable [23,25]. Clinical metadata are minimal, with no treatment status, disease duration, erosive versus reticular subtype, smoking, hepatitis C status or follow-up recorded, so clinical heterogeneity, therapeutic response and outcome cannot be modelled, and the erosive/reticular distinction that carries much of the clinical signal in OLP [93,94] is invisible here. The genital arm comprises female vulvar samples only, and sex is confounded with site in that comparison despite adjustment in the oral model. Finally, LP diagnostic criteria and biopsy handling differ across the source studies, and hypertrophic and classic cutaneous lesions were pooled after finding no differentially expressed genes between them, which limited power does not allow us to interpret as biological identity. Adequately powered, compartment-matched, single-cell or spatial studies of oral, genital and cutaneous LP within one protocol are the necessary next step [11,70,95], together with organotypic models in which individual barrier genes can be perturbed [65].

## 4. Materials and Methods

### 4.1. Dataset Identification and Inclusion

The Gene Expression Omnibus [96,97] was searched for human LP expression series with lesional and control samples and platform annotation. Four series met criteria and were analysed in full (Table 1): GSE52130, oral and genital LP epithelium and matched normal epithelium on Illumina HumanHT-12 v4 (*n* = 23; oral 7 LP/7 control, genital 5 LP/4 control; sex annotated) [98]; GSE130403, lesional classic (*n* = 21) and hypertrophic (*n* = 17) cutaneous LP skin and normal skin (*n* = 12) on Affymetrix HuGene 2.1 ST [8]; GSE38616, an independent oral cohort (7 OLP/7 control) on HuGene 1.0 ST; and GSE38615, microRNA arrays from the same 14 biopsies [39]. Series GSE70664–GSE70666 were retrieved but excluded (*n* = 2 arrays each, no replication possible).

Two departures from previous analyses of these data are material. First, the full GSE130403 series (50 arrays) was used, whereas the source publication analysed a 7-versus-7 subset; second, the genital arm of GSE52130 was retained, providing a second mucosal site on the discovery platform. All arrays are bulk microarrays; no single-cell or spatial data were generated or reanalysed here, and single-cell findings are used only as external context in the Discussion.

### 4.2. Preprocessing and Gene Annotation

Series matrices were downloaded programmatically. Platform annotation files (GPL10558, GPL17692, GPL6244, GPL8786) were used to map probes to HGNC symbols; multi-symbol and unannotated probes were discarded. Where several probes mapped to one gene, the probe with the highest mean expression was retained. Values already log-transformed by the submitter were used as provided; the microRNA matrix of GSE38615 was on a linear scale and was log_2_(x + 1)-transformed. Genes were retained if expression exceeded the dataset median in at least 30% of samples, giving 10,920 (GSE52130 oral), 10,573 (GSE52130 genital), 15,575 (GSE130403) and 12,437 (GSE38616) analysable genes.

### 4.3. Quality Control and Batch Assessment

For each cohort, principal-component analysis was performed on the filtered matrix; the variance explained by the first five components, the mean inter-array correlation and per-array correlation outliers (mean correlation more than three median absolute deviations below the cohort median) were recorded. The association of PC1–PC3 with disease status and with available covariates (sex, LP subtype) was tested by analysis of variance so that structure attributable to technical or demographic factors was identified before differential testing (Table S2).

### 4.4. Differential Expression with Covariate Adjustment

Differential expression used limma-style linear models with empirical Bayes moderation, implemented in InMoose, the Python port of limma [99,100,101]. Genes were considered differentially expressed at Benjamini–Hochberg false discovery rate (FDR) < 0.05 and |log_2_ fold change| ≥ 1 [102]. The oral model included sex as a covariate; the unadjusted model was fitted in parallel to quantify the effect of adjustment. For cutaneous LP, three contrasts were fitted (classic versus normal, hypertrophic versus normal, hypertrophic versus classic) in addition to all-LP versus normal.

### 4.5. Cross-Platform Integration: Meta-Analysis and Batch Correction

The two oral cohorts were combined by two independent strategies. First, a random-effects inverse-variance meta-analysis was computed on the 8106 genes measured in both series, with per-gene standard errors derived from the moderated statistics, between-study variance estimated by DerSimonian–Laird, and heterogeneity summarised by Cochran’s Q and I^2^. Second, the two matrices were merged on shared genes (18,442 genes, 28 samples) and batch-corrected with ComBat [103,104], with success judged by the association of PC1 with platform before and after correction, and differential expression then refitted on the corrected matrix. Agreement between the two strategies was quantified by the Jaccard index of their significant gene sets.

### 4.6. Sensitivity Analyses

Three sensitivity analyses addressed sample-size imbalance and influential observations. (i) The cutaneous cohort was randomly down-sampled to 7 LP versus 7 controls, matching the discovery cohort size, and the analysis was refitted. (ii) Leave-one-out analysis removed each sample in turn (14 iterations in the oral cohort, 50 in the cutaneous cohort) and recorded how often each hub gene retained significance. (iii) Cutaneous subtype contrasts tested whether classic and hypertrophic lesions could be pooled.

### 4.7. Shared Signature, Site Divergence and the Interaction Model

Shared genes were defined as those significant in both the oral and the cutaneous discovery cohort; direction of effect was recorded rather than imposed, so the shared set contains both concordant and discordant genes. Concordant shared genes were then assigned evidence tiers by the number of independent cohorts (oral discovery, cutaneous, genital, independent oral) in which they were significant, with Tier 1 requiring all four.

Site divergence was assessed at two levels. As a descriptive analysis, oral (meta-analytic) and cutaneous effect sizes were compared for all commonly tested genes by Spearman correlation and by a two-sample z-test on the difference of log_2_ fold changes with BH correction. Because this comparison is confounded by platform, laboratory and biopsy compartment, the primary divergence analysis was an interaction model fitted within GSE52130 alone, that is, one platform, one laboratory and one submitter, testing gene-wise disease × site (oral versus genital) interaction, so that divergence between two mucosal sites is estimated without cross-study confounding.

### 4.8. Pathway Activity

Single-sample gene set enrichment scores for the 50 MSigDB hallmark sets [105,106,107] were computed for every array with GSEApy [108]. Differential pathway activity per cohort was tested by moderated linear models on the score matrix, and differential activity between oral and cutaneous LP was tested on the cohort-wise activity differences. Over-representation analysis of gene lists used Reactome and hallmark libraries through Enrichr [109,110,111,112].

### 4.9. Immune and Stromal Composition, and the Compartment Proxy

Immune and stromal abundance was estimated with MCP-counter marker sets [113] and compared between lesional and control samples within each cohort. To test whether between-cohort differences in inferred infiltrate reflect the tissue compartment sampled rather than disease intensity, a compartment proxy was computed for every cohort as the mean within-cohort expression rank of keratinocyte-differentiation markers and of pan-immune markers; a cohort profiling separated epithelium is expected to show a high keratinocyte and a low immune rank, whereas full-thickness tissue is expected to show similar ranks for both. The genes in each marker set are listed in Table S36A. Sensitivity of the proxy to marker choice was assessed with (i) a basal and structural keratinocyte set chosen to exclude cornification genes that are themselves altered in LP; (ii) a T-lymphoid-only immune set; (iii) the ESTIMATE immune and stromal signatures [29]; and (iv) 1000 random 70% subsamples of the marker sets. ESTIMATE scores were additionally computed by single-sample gene set enrichment analysis [105] with GSEApy [108], as in the original method, and compared in control samples across the LP and comparator series, because control tissue reflects biopsy depth without the effect of the lesional infiltrate.

### 4.10. Protein–Protein Interaction Network

Shared genes were mapped to the STRING human network [114] using high-confidence interactions only (combined score ≥ 700). STRING returns current preferred gene names, which differ from the array-annotation symbols for some genes (for example, HIST1H2AC/H2AC6); returned names were therefore mapped back to the queried symbols before graph construction, so that the network contains exactly the 85 shared genes and no gene appears twice under two aliases. Degree, betweenness and eigenvector centrality were computed with NetworkX, and modules were detected by greedy modularity optimisation. Because the cornified-envelope gene families are physically clustered and functionally interchangeable, hub status of individual LCE and SPRR paralogues was interpreted as a property of the family module rather than of single genes. NetworkX is a deterministic graph-analysis library and contains no machine-learning or artificial-intelligence components.

### 4.11. Barrier Score and Cross-Cohort Evaluation

A barrier score was defined as the mean z-score of the up-regulated Tier 1 and Tier 2 genes (52 genes) and computed per sample in each cohort. Discrimination between lesional and control samples was summarised by the area under the receiver-operating-characteristic curve (AUC) with Mann–Whitney U tests. Because the panel was derived from the oral and cutaneous discovery cohorts, the AUCs obtained in those two cohorts are in-sample estimates and are circular by construction. The genital arm of GSE52130 was not used for derivation and therefore provides an out-of-derivation estimate, although it shares a study and platform with the oral discovery arm. The independent oral cohort GSE38616 is the only fully independent cohort and provides the strictest transfer estimate. Estimates are reported and interpreted in that order. To obtain non-circular estimates, panels were also re-derived from a single discovery cohort, using genes annotated to the Reactome Keratinization or Formation of the Cornified Envelope pathways, plus KPRP (218 genes), that were up-regulated at FDR < 0.05 with log_2_FC ≥ 1, and scored in every other cohort; an a priori score of all measured LCE and SPRR family genes required no derivation. In GSE38616, the observed AUC was further assessed against 5000 random gene sets matched to the measurable panel genes by expression-level ventile, by a stratified bootstrap 95% confidence interval (5000 resamples), and by a binomial test of direction concordance across signature genes.

### 4.12. MicroRNA Analysis

MicroRNA differential expression was computed in GSE38615 with the same modelling framework. To test the previously proposed microRNA axis, per-sample barrier scores computed from the matched mRNA arrays of the same 14 biopsies (GSE38616) were correlated with per-sample microRNA expression by Spearman correlation, and individual microRNA–target pairs (including miR-20a-5p and miR-34a-5p against the barrier score and against individual barrier genes) were tested with BH correction across all tested microRNAs.

To test the proposed regulatory axis directly rather than through network topology, we then queried experimentally supported microRNA–target interactions for every nominally differentially expressed microRNA (with miR-20a-5p and miR-34a-5p added irrespective of significance) through the multiMiR interface [115] to TarBase v9.0 [116] and miRTarBase 2025 [117], retaining only records with reported experimental support and discarding purely predicted interactions; both -3p and -5p mature forms of each array probe were matched to the interaction tables. Every supported microRNA–target pair whose gene was measured on the matched mRNA arrays of the same 14 biopsies was tested by Spearman correlation across those biopsies, with BH correction across all tested pairs, and a pair was scored as directionally consistent with canonical repression only when the correlation was negative and the microRNA and its target moved in opposite directions in the lesional-versus-control comparison. The analysis was then restricted to the 83 concordant signature genes and the correlation distributions of signature and non-signature pairs were compared by Mann–Whitney U test. Finally, for the microRNAs implicated in this test, we searched the primary literature for independent lichen planus tissue evidence, keeping cutaneous LP skin and oral LP mucosa separate.

### 4.13. Protein-Level Verification and Reproducibility

Tier 1 and Tier 2 genes were checked against Human Protein Atlas tissue records for documented protein expression in oral mucosa and skin [63,118]. All analyses were performed in Python 3.14; the complete, ordered analysis scripts, the software versions and every derived table are provided so that the entire study can be re-executed from the public raw data (Data Availability).

### 4.14. Comparator Datasets and Specificity Analysis

Public series of other chronic inflammatory skin and mucosal diseases with lesional and control samples were selected to span psoriasiform, interface, eczematous and mucosal inflammation: GSE13355 (psoriasis, 58 lesional versus 64 normal skin samples from healthy controls; Affymetrix U133 Plus 2.0) [48]; GSE109248 (discoid, subacute and acute cutaneous lupus, 6, 12 and 7 lesions; psoriasis, 17 lesions; normal skin, 14; Illumina HumanHT-12 v4) [47]; GSE32924 (atopic dermatitis, 13 lesional versus 8 normal; U133 Plus 2.0) [26]; GSE63741 (LP, psoriasis, atopic dermatitis and contact eczema, 30 each, and 30 healthy controls; two-colour skin-focused array against a common reference) [46]; GSE228083 (eosinophilic oesophagitis, 12 versus 12; Clariom S) [49]; and GSE10334 (periodontitis, 183 diseased versus 64 healthy gingival sites; U133 Plus 2.0) [50]. Uninvolved skin from patients was not used as a control. Series matrices were processed as in Section 4.2, and the 52-gene barrier score and the 83-gene signature were applied without modification. Differential expression used the same moderated linear models, and a signature gene was considered concordantly changed in a comparator when it reached FDR < 0.05 in the same direction as in LP. Panel genes were classified as LP-restricted (concordant in no comparator), shared with psoriasis only, or shared more broadly. LCE1/LCE2 (LCE1A–F, LCE2A–D) and LCE3 (LCE3A–E) group scores were computed as mean z-scores and summarised by AUC and by the standardised mean difference (lesional minus control, divided by the pooled standard deviation). Re-implementation of the barrier score from the public matrices reproduced the published LP AUCs to within 0.01.

## 5. Conclusions

Full reanalysis of all eligible public LP expression series identified by our search supports a single, reproducible conclusion: oral, genital and cutaneous LP share a replication-graded epithelial barrier and cornified-envelope programme, of which only BPIFC, CDSN and WFDC12 replicate in all four cohorts examined. This programme transfers between the two discovery studies but did not reach significance in the small independent oral cohort, and it is shared with psoriasis and partly with cutaneous lupus, so it is a common feature of the LP spectrum rather than a marker specific to LP. Differences in interferon and immune-cell signal between mucosal and cutaneous series are strongly influenced by, and cannot be separated from, differences in the tissue compartment sampled, and should not be read as site-specific biology, whereas the genuine divergence measurable within one study, between oral and genital mucosa, involves oestrogen-response and regional keratinisation programmes. MicroRNAs nominated from network topology alone, including miR-20a-5p and miR-34a-5p, are not supported by matched tissue microRNA data, and experimentally supported microRNA–target pairs involving the shared programme correlate positively rather than inversely, so a microRNA-repression axis linking lesional microRNAs to barrier-gene expression is not detectable in these tissues. Barrier disruption is therefore the most defensible shared molecular feature of the mucocutaneous LP spectrum, though not one unique to it, and the appropriate object of the compartment-matched, experimentally validated studies that must follow.

## Figures and Tables

**Table 1:** Datasets reanalysed in this study.

Accession	Platform	Tissue	Groups (*n*)	Use in This Study
GSE52130	Illumina HumanHT-12 v4 (GPL10558)	Oral and vulvar mucosal epithelium (separated)	Oral LP 7/control 7; genital LP 5/control 4	Oral discovery cohort; genital cohort; within-study site interaction model
GSE130403	Affymetrix HuGene 2.1 ST (GPL17692)	Lesional skin, full thickness	Classic LP 21; hypertrophic LP 17; normal 12	Cutaneous cohort; subtype contrasts; down-sampling and leave-one-out
GSE38616	Affymetrix HuGene 1.0 ST (GPL6244)	Oral mucosa	OLP 7/control 7	Independent oral cohort for targeted replication and barrier-score transfer
GSE38615	Affymetrix miRNA array (GPL8786)	Oral mucosa (same 14 biopsies as GSE38616)	OLP 7/control 7	Matched microRNA data for the microRNA-axis test

## Data Availability

All primary data are publicly available from the Gene Expression Omnibus under accessions GSE52130, GSE130403, GSE38615, and GSE38616; comparator series were obtained under accessions GSE13355, GSE109248, GSE32924, GSE63741, GSE228083, and GSE10334. Experimentally supported microRNA–target interactions were obtained from TarBase v9.0 and miRTarBase 2025 through multiMiR. All derived result tables are provided as Supplementary Tables S1–S37, and the complete analysis code, executed in order with recorded software versions, is provided as a supplementary code package.

## Supplementary Materials

The following supporting information can be downloaded at: https://www.mdpi.com/article/10.3390/ijms27219534/s1.

## Abbreviations

The following abbreviations are used in this manuscript:

LP	lichen planus
OLP	oral lichen planus
AUC	area under the receiver operating characteristic curve
BH	Benjamini and Hochberg
DEG	differentially expressed gene
FDR	false discovery rate
GEO	Gene Expression Omnibus
GSVA	gene set variation analysis
HPA	Human Protein Atlas
LCE	late cornified envelope
PCA	principal component analysis
PPI	protein–protein interaction
ROC	receiver operating characteristic
SPRR	small proline-rich protein
